# Sleep duration moderates association between screen time and emotional and behavioural problems in young children

**DOI:** 10.1007/s12519-025-00963-x

**Published:** 2025-09-23

**Authors:** Lucía Iglesias-Vázquez, Tany E. Garcidueñas-Fimbres, Carlos Gómez-Martínez, Cristina Castro-Collado, Rosaura Leis, María Fernández de la Puente, Luis A. Moreno, Santiago Navas-Carretero, Dolores Corella, Ana Moreira Echeverria, José M. Jurado-Castro, Rosaura Picáns-Leis, Jiaqi Ni, Maria L. Miguel-Berges, J. Alfredo Martínez, María I. Benedicto-Toboso, Francisco Llorente-Cantarero, Rocío Vázquez-Cobela, Albert Feliu, Guiomar Masip, Belén Pastor-Villaescusa, Mercedes Gil-Campos, Joaquín Escribano, Nancy Babio

**Affiliations:** 1 Universitat Rovira I Virgili, Departament de Bioquímica I Biotecnologia, Alimentació, Nutrició, Desenvolupament i Salut Mental (ANUT-DSM), Unitat de Nutrició Humana, Sant Llorenç, 21, 43201 Reus, Spain; 2 Institut d’Investigació Sanitària Pere Virgili (IISPV), Carrer Dr. Mallafré Guasch, 4, 43007 Tarragona, Spain; 3 Consorcio CIBER, M.P. Fisiopatología de la Obesidad y Nutrición (CIBEROBN), Instituto de Salud Carlos III (ISCIII), Av. Monforte de Lemos, 3-5. Pabellón 11. Planta 0, 28029 Madrid, Spain; 4 Department of Epidemiology and Public Health, Hospital de la Santa Creu i Sant Pau. Biomedical Research Institute Sant Pau (IIB Sant Pau), Sant Quintí, 89, Barcelona, Spain; 5 Division of Psychiatry, Haukeland University Hospital, 5021 Bergen, Norway; 6 Department of Biomedicine, University of Bergen, 5009 Bergen, Norway; 7 University of Córdoba, Metabolism and Investigation Unit, Reina Sofia University Hospital, Maimónides Institute of Biomedicine Research of Córdoba (IMIBIC), Av. Menéndez Pidal, S/N, 14004 Córdoba, Spain; 8 Unit of Pediatric Gastroenterology, Hepatology and Nutrition, Pediatric Service, Hospital Clínico Universitario de Santiago, Rúa da Choupana, S/N, 15706 Santiago de Compostela, A Coruña, Spain; 9 Pediatric Nutrition Research Group, Unit of Investigation in Nutrition, Growth and Human Development of Galicia-USC, Health Research Institute of Santiago de Compostela (IDIS), Rúa da Choupana, S/N, 15706 Santiago de Compostela, A Coruña, Spain; 10 Growth, Exercise, Nutrition and Development (GENUD) Research Group and Instituto Agroalimentario de Aragón (IA2), University of Zaragoza, Pedro Cerbuna, 12, 50009 Zaragoza, Spain; 11 Instituto de Investigación Sanitaria de Aragón (IIS Aragón), Centro de Investigación Biomédica de Aragón (CIBA). Avda. San Juan Bosco, 13, 50009 Zaragossa, Spain; 12 Center for Nutrition Research and Fac Pharm & Nutr, Dept Nutr Food Sci & Physiol, University of Navarra, Irunlarrea, 1, 31008 Pamplona, Spain; 13 IdisNA, Navarra Institute for Health Research, Irunlarrea, 3, 31008 Pamplona, Spain; 14 Department of Preventive Medicine and Public Health, University of Valencia. Av. de Blasco Ibáñez, 13, El Pla del Real, 46010 Valencia, Spain; 15 Fundació Hospital Sant Joan de Déu de Martorell, Av. Mancomunitats Comarcals, 1, 3, 08760 Martorell, Barcelona, Spain; 16 Nutrición de Precisión y Programa de Salud Cardio Metabólica, Instituto Madrileño de Estudios Avanzados (IMDEA), Consejo Superior de Investigaciones Científicas-Campus de Excelencia Internacional (CSIC-CEI), 28049 Madrid, Spain; 17 Hospital Universitari Salut Sant Joan de Reus; and Pediatric Nutrition and Human Development Research Unit, Universitat Rovira I Virgili, Sant Llorenç, 21, 43201 Reus, Spain

**Keywords:** Child, Mental health, Screen time, Sleep, Strengths and difficulties questionnaire

## Abstract

**Background:**

Preschoolers and young children are vulnerable to psychosocial and behavioral disorders linked to lifestyle factors such as screen time and sleep disturbances. Our study examines the relationship between screen time and adherence to recommendations with children’s behavioral and emotional difficulties, with a focus on the role of sleep duration.

**Methods:**

Cross-sectional analyses were conducted within the multicenter prospective Childhood Obesity Risk Assessment Longitudinal Study (CORALS), which included 1420 children aged 3–6 years. Screen time (hours/day) and adherence to recommendations (≤ 2 hours/day) were assessed. Behavioral and emotional difficulties were measured via the strengths and difficulties questionnaire. Multivariable linear and logistic regression models were used to estimate associations between screen time (continuous and dichotomous) and strengths and difficulties questionnaire scores, adjusting for potential confounders. We also tested the moderating effect of sleep and conducted isotemporal substitution analyses replacing screen time with sleep duration.

**Results:**

Higher screen time was associated with higher total strengths and difficulties questionnaire scores [*β* 95% confidence interval (CI), 0.35 (0.10, 0.61)], emotional symptoms [0.10 (0.01, 0.19)], conduct problems [0.10 (0.01, 0.18)], and greater odds of exceeding the 16-point strengths and difficulties questionnaire cutoff for behavioral and emotional difficulties [odds ratio (OR) (95% CI), 1.21 (1.04, 1.41)]. Children who adhered to screen time recommendations had lower strengths and difficulties questionnaire total scores [*β* (95% CI), − 0.64 (− 1.19, − 0.10)] and odds of experiencing behavioral and emotional difficulties [OR (95% CI), 0.67 (0.47, 0.95)]. Sleep duration moderated the screen time–strengths and difficulties questionnaire association (*P* = 0.020). The isotemporal substitution of screen time for sleep duration was associated with lower strengths and difficulties questionnaire scores across all subscales, except for prosocial behavior.

**Conclusions:**

Higher screen time was associated with greater emotional and behavioral difficulties, whereas adherence to screen time recommendations and adequate sleep duration were inversely associated. Managing screen time and promoting sleep are crucial for children’s well-being.

**Graphical abstract:**

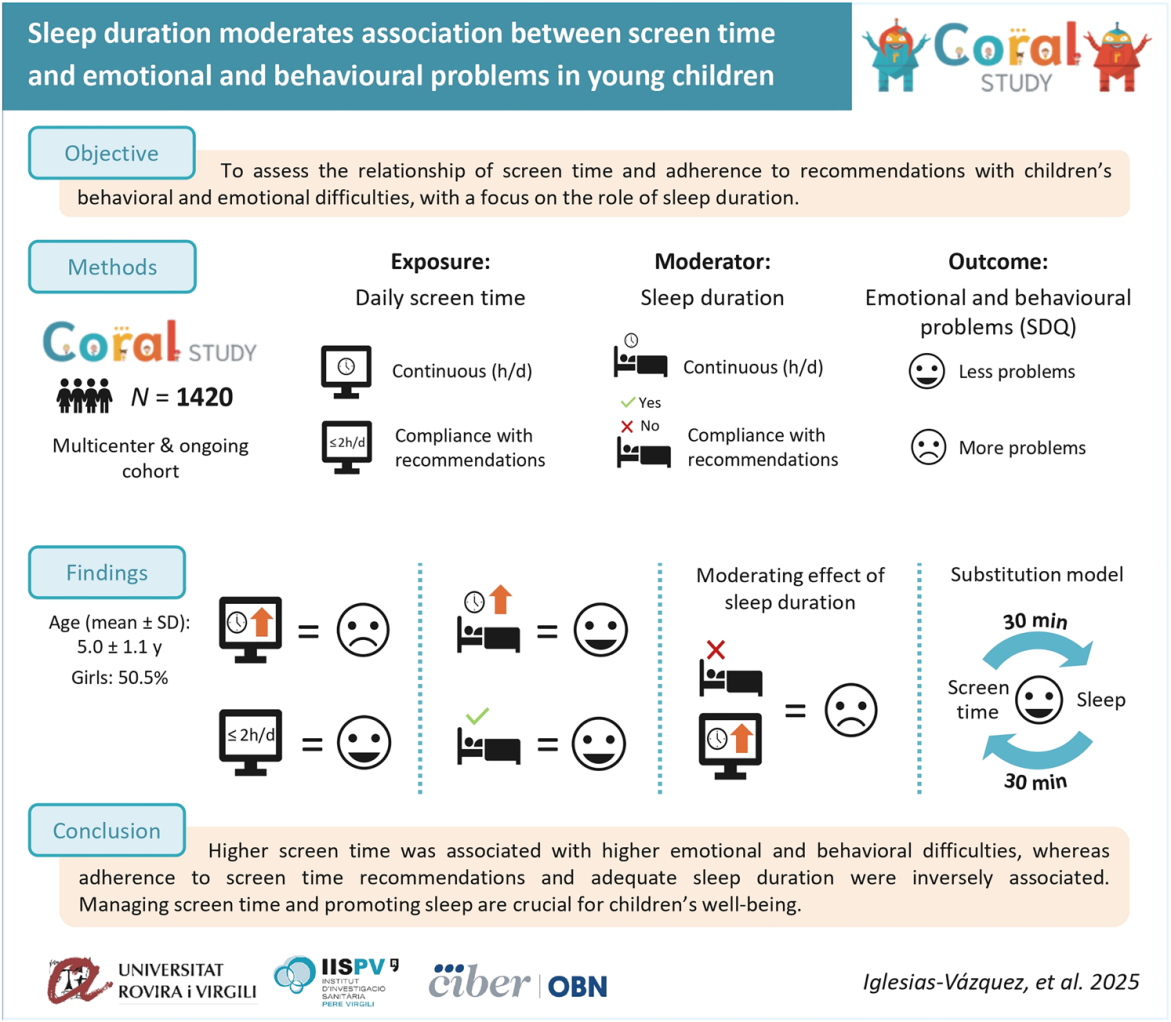

**Supplementary Information:**

The online version contains supplementary material available at 10.1007/s12519-025-00963-x.

## Introduction

Mental health is a fundamental component of well-being and a basic human right [1]. Childhood and adolescence are critical life stages characterized by significant brain growth and development [2]. A recent United Nations International Children's Emergency Fund (UNICEF) report highlights an alarming prevalence of mental disorders among children and adolescents aged 10–19 years in Western Europe, with a prevalence of about 16% in boys and 17% in girls, compared with a global prevalence of 13% [2]. Specifically, in Spain, the rate of psychiatric diagnoses in adolescents significantly raised from 3.9% in 2000 to 9.5% in 2021 [3]. While certain mental health disorders are more common in adolescents, behavioral and emotional difficulties, including hyperactivity, conduct issues, and emotional dysregulation, may also emerge in younger children and persist into adulthood [4–7]. This underscores the importance of early identification and prevention strategies to mitigate potential long-term consequences [8].

Despite this, most research has focused on adolescents, with relatively few studies examining the early childhood period. The evidence suggests that preschoolers and young children are also vulnerable to psychosocial and behavioral challenges linked to lifestyle factors such as screen time and sleep disturbances [8–10]. Several studies and systematic reviews have reported associations between excessive screen time and adverse developmental outcomes in children, including poorer mental health, sleep problems, cognitive delays, and behavioral difficulties [5, 8–10]. Nonetheless, the extant literature is controversial, with some studies reporting significant relationships and others finding weak, inconsistent, or null associations, highlighting the need for further high-quality research in this area [11]. Consequently, international health organizations, including the World Health Organization (WHO) [12], the American Academy of Child and Adolescent Psychiatry [13], and the American Academy of Pediatrics [14], recommend limiting screen exposure in children and adolescents as part of broader preventive health strategies.

In addition to the potential direct impact of screen time on mental health, certain reviews have suggested that the COVID-19 pandemic has contributed to increases in screen time and mental health problems in children and adolescents [15, 16]. In addition, both child lifestyle factors (including diet and sleep duration) and parental factors (including socioeconomic status) appear to play important roles in moderating this relationship [7, 17–19]. Some studies have reported that greater screen exposure is associated with shorter sleep duration, later sleep onset, and reduced sleep quality [6, 18, 20]. Moreover, children who do not meet sleep duration recommendations are particularly susceptible to the negative effects of screen time on mental health [18]. However, the literature remains mixed, and meta-analyses indicate that these associations are generally small in magnitude [11, 21].

The present study aimed to examine the relationship between screen time and behavioral and emotional difficulties in children, as assessed via the strengths and difficulties questionnaire (SDQ), with a particular focus on the role of sleep duration. We hypothesized that greater screen time and noncompliance with screen time recommendations would be associated with greater emotional and behavioral difficulties, with sleep duration potentially playing an important role in these associations. By analyzing these relationships in a large multicenter cohort of 3–6-year-old children in Spain, we aim to contribute to the growing body of evidence supporting strategies that promote healthy screen use and sleep habits in early childhood.

## Methods

### Study design and participants

A cross-sectional study design was used within the Childhood Obesity Risk Assessment Longitudinal Study (CORALS) cohort. The CORALS project is a 10-year ongoing multicenter prospective study conducted in Spain (https://corals.es) aiming to identify risk factors for childhood obesity. Eligible participants were children aged 3–6 years who were recruited between March 22, 2019, and June 30, 2022 and who attended selected schools in seven Spanish cities. The exclusion criteria included children from families with difficulties complying with the study protocol, specifically, the inability or unwillingness to provide written informed consent, the inability to communicate with the study staff, illiteracy, difficulties with comprehension or language, or unstable domiciles.

Among the 1509 CORALS participants, 1420 with complete data for screen time and SDQ were analyzed.

### Screen time

Screen time was measured by first asking parents to report how many hours per day their child spent watching television and then spent playing video games on a computer, mobile phone, or game console. Additionally, each of these questions asked about the time spent on weekdays and weekends.

The reported weekday hours were multiplied by five, and the weekend hours were multiplied by two. Then, both totals were added together and divided by seven to obtain the average daily screen time. Screen time was assessed both continuously (hours/day) and dichotomously as compliance with screen time recommendations (compliance; noncompliance). For the main analyses, which included the entire population of children aged 3–6 years (*n* = 1240), screen time compliance was defined according to the American Academy of Pediatrics (AAP) recommendation of ≤ 2 hours/day [14]. Additionally, secondary analyses were restricted to participants aged 3–4 years (*n* = 737), in accordance with the WHO recommendation of ≤ 1 hour/day for children under five years of age [22].

### Sleep duration

Parents were asked how many hours per day their children slept at night and during naps, both on weekdays and weekends, in separate questions. Sleep duration (hours/day) and the degree of sleep duration (inadequate; adequate) were obtained. An adequate sleep pattern was considered when it ranged between 10 and 14 hours/day for children under six years old and between nine and 12 hours/day for children over six years old. Sleeping less or more than these values was considered an inadequate sleep pattern [23].

### Emotional and behavioral strengths and difficulties

The SDQ was completed by parents or caregivers. The SDQ is a screening tool developed to assess the emotional and behavioral strengths and difficulties of children and adolescents [24]. The total SDQ score (including all the subscales except the prosocial behavior subscale) ranges from 0 to 40, with higher scores indicating greater emotional and behavioral difficulties. A total SDQ score of 16 or 17 was suggested to indicate a borderline or abnormal risk for potential mental health problems, respectively [25, 26]. The SDQ has been validated in the Spanish population, indicating acceptable reliability estimates [24]. In our study, the total SDQ score also showed acceptable internal consistency (Cronbach’s alpha = 0.75). Details about the SDQ are provided in the Supplementary Methods.

### Assessments and covariates

Self-administered questionnaires were provided to parents or caregivers, and data on the sociodemographic, lifestyle, and early-life factors, as well as paternal and maternal factors, of the children were collected. Anthropometric variables were measured by trained personnel. A full description of the covariates and their coding is provided in the Supplementary Methods.

### Statistical analysis

The CORALS database, updated to February 2024, was used. The general characteristics are reported as the means ± standard deviations (SDs) for numerical variables and as numbers (percentages) for categorical variables. T tests and chi-square tests were performed to compare general characteristics by adherence to screen time recommendations. Information about missing data and imputation is provided in the Supplementary Methods.

Linear regression models, using *β* coefficients and 95% confidence intervals (95% CIs), were fitted to assess associations between screen time (continuously and dichotomously with the AAP cutoff) and SDQ scores, and Cohen’s d effect size was estimated to evaluate the magnitude of observed group differences. Logistic regression models, using odds ratios (ORs) and 95% CIs, were used to evaluate the relationships between screen time (continuously and dichotomously with the AAP cutoff) and the odds of presenting behavioral and emotional difficulties, using both the 16- and 17-point SDQ cutoffs. Noncompliance with screen time recommendations served as the reference category. As a secondary analysis, linear regression models were used to assess the association between screen time (dichotomously with the WHO cutoff) and the total SDQ score for children under five years of age. The models were adjusted for center, sex, age, body mass index (BMI) z score, physical activity, exclusive breastfeeding, and both maternal and paternal BMI, educational level, and socioprofessional status. All reported *β* coefficients are unstandardized, meaning that both the exposure (screen time) and the outcome (SDQ scores) were analyzed in their original measurement units.

Linear regression models were used to assess the associations between sleep duration (continuously and dichotomously) and the total SDQ score. Additionally, the potential moderating effect between the adequacy of sleep duration and screen time on the total SDQ was assessed via the likelihood ratio test, which compared the models with and without the interaction product of screen time and sleep duration. If a statistically significant interaction was detected, the results were stratified according to sleep duration recommendations (inadequate; adequate). Additionally, simulated isotemporal substitution models were used to estimate the potential effect of replacing 30 minutes per day of screen time with an equal amount of sleep while holding the total time constant. This approach assumes that the time spent in daily activities is finite and substitutable and allows the modeling of trade-offs between behaviors. A 30-minute time block was selected because it represents a meaningful and interpretable unit of change in children’s daily routines.

For sensitivity analyses, interaction analyses using the likelihood ratio test were conducted to explore the associations between screen time and total SDQ according to whether participants were assessed during the presence or absence of COVID-19 sanitation restrictions in Spain (March 14, 2020, to June 30, 2021) [27, 28], as were participants’ age, sex, adherence to the Mediterranean diet (MedDiet), weight status, maternal and paternal weight status, education level, and employment status.

Statistical analyses were performed via Stata 17 (StataCorp), with significance set at *P* < 0.05.

## Results

Of the 1509 participants who attended the CORALS baseline visit, 67 and 22 participants were excluded because of missing data on screen time and SDQ, respectively, resulting in a total population of 1420 children being analyzed (Supplementary Fig. 1). The participants had a mean age of about five years and an average screen time of 1.8 hours/day. About 10%–12% of the children scored above the SDQ cutoff thresholds for emotional and behavioral difficulties. Mothers completed questionnaires for 87% of the participants.

As shown in Table 1, children who met the screen time recommendations had significantly lower scores on the emotional and behavioral subscales of the SDQ, except for the prosocial subscale. These children presented longer sleep durations and adequate sleep patterns, greater adherence to the Mediterranean diet (MedDiet), a greater likelihood of being exclusively breastfed for the first six months, and lower BMI z scores and a higher prevalence of overweight/obesity. Both parents of children in the compliance category were younger, had a lower BMI and healthier weight status, and had higher education and socioprofessional status (Supplementary Table 1).

**Table 1:** General characteristics of the study population according to compliance with screen time recommendations

Variables	All population *n* = 1420	Categories of compliance with screen time recommendations (≤ 2 h/d)		
		Non-compliance *n* = 452 (> 2 h/d)	Compliance *n* = 968 (≤ 2 h/d)	*P*-value^**a**^
Sociodemographic characteristics				
Age (y)	4.97 ± 1.11	5.31 ± 1.05	4.82 ± 1.11	< 0.001
Girls, *n* (%)	717 (50.49)	222 (49.12)	495 (51.14)	0.478
Lifestyle behaviours				
Screen time, h/d	1.80 ± 1.05	3.01 ± 0.89	1.24 ± 0.49	< 0.001
Total sleep duration, h/d	10.39 ± 0.90	10.23 ± 0.92	10.47 ± 0.88	< 0.001
Sleeping pattern for age				< 0.001
Adequate, *n* (%)	1153 (81.20)	341 (75.44)	812 (83.88)	
Physical activity, minutes/week	190.61 ± 114.32	188.82 ± 110.05	191.44 ± 116.31	0.688
Healthy behaviour (≥ 120 min/wk), *n* (%)	1016 (71.55)	323 (71.46)	693 (71.59)	0.959
Adherence to the MedDiet, 0–18 points	10.79 ± 2.76	10.23 ± 2.62	11.05 ± 2.75	< 0.001
Anthropometry				
zBMI	0.00 ± 1.00	0.17 ± 1.17	− 0.08 ± 0.90	< 0.001
Weight status				< 0.001
Underweight or normal weight, *n* (%)	1127 (79.37)	327 (72.35)	800 (82.64)	
Overweight or obesity, *n* (%)	293 (20.63)	125 (27.65)	168 (17.36)	
COVID-19 sanitary restrictions in Spain				
Participants recruited during the restrictions (May 14, 2020–June 30, 2025)	1122 (79.01)	87 (19.25)	211 (21.80)	0.272
Emotional and behavioural assessment				
Total SDQ score, 0–40 points^**b**^	9.95 ± 4.75	10.67 ± 4.83	9.61 ± 4.68	< 0.001
Emotional symptoms scale, 0–10 points	1.81 ± 1.67	2.03 ± 1.68	1.70 ± 1.65	< 0.001
Conduct problems scale, 0–10 points	2.23 ± 1.62	2.37 ± 1.67	2.17 ± 1.60	0.032
Hyperactivity-inattention scale, 0–10 points	4.61 ± 2.42	4.80 ± 2.33	4.52 ± 2.46	0.046
Peer problems scale, 0–10 points	1.30 ± 1.45	1.48 ± 1.50	1.22 ± 1.41	0.002
Prosocial behaviour scale, 0–10 points^**c**^	8.11 ± 1.68	8.07 ± 1.69	8.12 ± 1.68	0.595
Total SDQ score ≥ 16 points, *n* (%)	176 (12.39)	76 (16.81)	100 (10.33)	0.001
Total SDQ score ≥ 17 points, *n* (%)	132 (9.30)	57 (12.61)	75 (7.75)	0.003
Early life factors				
Birth weight, kg	3.25 ± 0.54	3.23 ± 0.54	3.26 ± 0.53	0.344
Birth weight				0.516
Low birth weight, *n* (%)	98 (6.90)	35 (7.74)	63 (6.51)	
Normal birth weight, *n* (%)	1231 (86.69)	385 (85.18)	846 (87.40)	
High birth weight, *n* (%)	91 (6.41)	32 (7.08)	59 (6.10)	
Total duration of breastfeeding				0.575
< 2 y, *n* (%)	1308 (92.11)	419 (92.70)	889 (91.84)	
≥ 2 y, *n* (%)	112 (7.89)	33 (7.30)	79 (8.16)	
Exclusive breastfeeding duration				0.014
< 6 mon, *n* (%)	832 (58.59)	286 (63.27)	546 (56.40)	
≥ 6 mon, *n* (%)	588 (41.41)	166 (36.73)	422 (43.60)	

Data are expressed as mean ± SD for continuous variables or number (percentage) for categorical variables

^**a**^*P*-values were calculated by t-test or chi-squared tests. ^b^The total SDQ score considers all subscales with the exception of the prosocial scale, with higher scores indicating higher overall levels of emotional and behavioural difficulties. ^c^Higher scores indicate higher prosocial capacities

*BMI* body mass index, *MedDiet* Mediterranean diet, *SD* standard deviation, *SDQ* strength and difficulties questionnaire

**Fig. 1:**
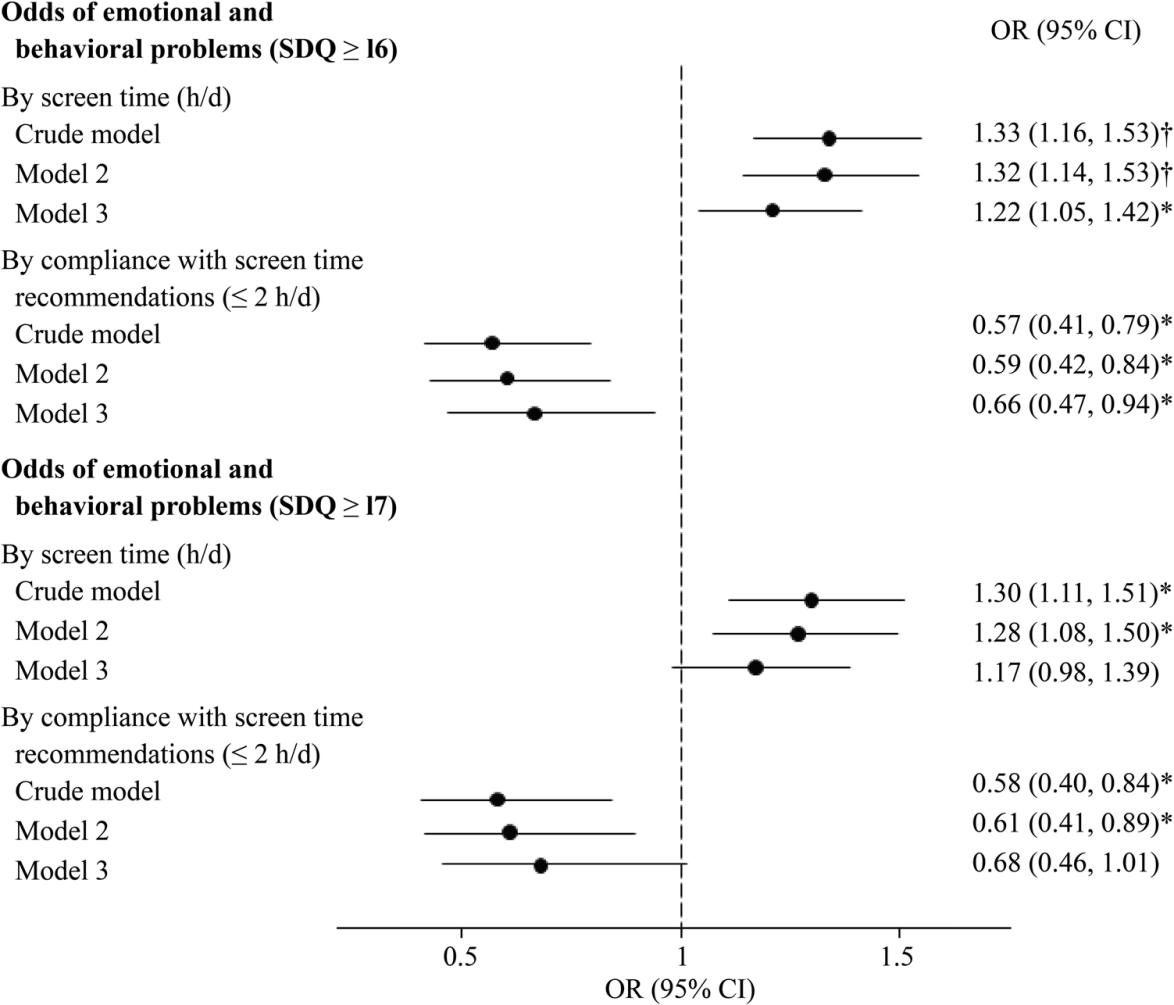
Associations between screen time and odds of emotional and behavioral problems. Logistic regression models, with ORs and 95% CIs, were fitted to assess the associations between screen time and the odds of developing emotional and behavioral problems. Crude model: unadjusted. Model 2: adjusted for center size (according to the number of participants: < 250, 250–400, > 400), sex, age (in years), zBMI, physical activity (minutes/week), and exclusive breastfeeding during the first six months (no; yes). Model 3: further adjusted for both maternal and paternal BMI (kg/m^2^), educational level (primary or lower; secondary; higher education), and socioprofessional status (homemaker/student/retired/unemployed; employed). * *P* value < 0.05; † *P* value < 0.001. *OR* odds ratio, *CI* confidence intervals, *SDQ* strengths and difficulties questionnaire, *BMI* body mass index, *zBMI* body mass index z-score

Table 2 shows that, in the fully adjusted models, higher screen time was associated with higher scores on the total SDQ and the emotional symptoms, conduct problems, and peer problems subscales. Associations with hyperactivity-inattention and prosocial behavior were not significant. Children adhering to screen time recommendations had significantly lower total SDQ, conduct and peer problems scores, although the effect sizes were small (Cohen’s *d* =  − 0.077). Secondary analyses revealed that children under five years of age who adhered to the screen time recommendation of ≤ 1 hour/day (*n* = 255) had lower total SDQ scores [− 0.86 (95% CI − 1.58, − 0.14), *P* = 0.019] than those who did not adhere to this recommendation. As illustrated in Fig. 1, greater screen time was associated with greater odds of behavioral and emotional difficulties, as indicated by the SDQ cutoff score of 16. In all these models, the mean variance inflation factor (VIF) values were less than 2.0, indicating that there was no multicollinearity.

**Table 2:** Associations between screen time and strength and difficulties questionnaire scores

Emotional and behavioural assessment	Screen time (h/d) *n* = 1420, *β* (95% CI)	Categories of compliance with screen time recommendations (≤ 2 h/d)	
		Non-compliance (> 2 h/d) *n* = 452, *β* (95% CI)	Compliance (≤ 2 h/d) *n* = 968, *β* (95% CI)
Total SDQ score^a^			
Crude model	0.64 (0.41, 0.87)†	0 (ref)	− 1.06 (− 1.59, − 0.54)†
Model 2	0.59 (0.34, 0.84)†	0 (ref)	− 0.94 (− 1.49, − 0.39)*
Model 3	0.39 (0.14, 0.64)*	0 (ref)	− 0.65 (− 1.20, − 0.10)*
Emotional symptoms scale^b^			
Crude model	0.19 (0.10, 0.27)†	0 (ref)	− 0.33 (− 0.52, − 0.15)†
Model 2	0.14 (0.05, 0.23)*	0 (ref)	− 0.22 (− 0.41, − 0.02)*
Model 3	0.11 (0.02, 0.20)*	0 (ref)	− 0.18 (− 0.37, 0.02)
Conduct problems scale^b^			
Crude model	0.10 (0.02, 0.18)*	0 (ref)	− 0.20 (− 0.38, − 0.02)*
Model 2	0.14 (0.05, 0.22)*	0 (ref)	− 0.28 (− 0.47, − 0.09)*
Model 3	0.10 (0.01, 0.19)*	0 (ref)	− 0.23 (− 0.42, − 0.04)*
Hyperactivity-inattention scale^b^			
Crude model	0.23 (0.11, 0.35)†	0 (ref)	− 0.27 (− 0.55, − 0.00)*
Model 2	0.20 (0.07, 0.32)*	0 (ref)	− 0.21 (− 0.49, 0.07)
Model 3	0.10 (− 0.03, 0.22)	0 (ref)	− 0.06 (− 0.34, 0.22)
Peer problems scale^b^			
Crude model	0.13 (0.06, 0.20)†	0 (ref)	− 0.26 (− 0.42, − 0.10)*
Model 2	0.12 (0.04, 0.20)*	0 (ref)	− 0.23 (− 0.40, − 0.06)*
Model 3	0.08 (0.00, 0.16)*	0 (ref)	− 0.18 (− 0.35, − 0.01)*
Prosocial behaviours scale^c^			
Crude model	− 0.00 (− 0.09, 0.08)	0 (ref)	0.05 (− 0.14, 0.24)
Model 2	− 0.04 (− 0.13, 0.05)	0 (ref)	0.14 (− 0.05, 0.34)
Model 3	− 0.05 (− 0.14, 0.04)	0 (ref)	0.16 (− 0.04, 0.36)

Linear regression models, with *β*-coefficients and 95% CI, were fitted to assess the associations between screen time and SDQ scores in the CORALS cohort

Crude model: unadjusted. Model 2: adjusted for centre size (according to the number of participants: < 200, 200–400, > 400), sex, age (in years), zBMI, physical activity (minutes/week), and exclusive breastfeeding during the first six months (no; yes). Model 3: further adjusted for both maternal and paternal BMI (kg/m^2^), educational level (primary or lower; secondary; academic/graduated), and socio-professional status (homemaker/student/retired/unemployed; employed)

^**a**^The total SDQ score considers all subscales with the exception of the prosocial scale, with higher scores indicating higher overall levels of emotional and behavioural difficulties. ^b^Higher scores indicate higher specific emotional and behavioural difficulties. ^c^Higher scores indicate higher prosocial capacities

* *P*-value < 0.05, †* P*-value < 0.001

*β (95% CI)* beta coefficients and 95% confidence intervals, *SDQ* strength and difficulties questionnaire, *BMI* body mass index, *zBMI* body mass index z-score, *ref *reference

A longer sleep duration was associated with a lower total SDQ score [*β* (95% CI), –0.46 (–0.74, –0.18); *P* = 0.001]. Compared with participants with an inadequate sleep pattern, those with an adequate pattern had a lower total SDQ score [–0.86 (–1.49, –0.24); *P* = 0.007]. The moderating effect between screen time and sleep duration on total SDQ was significant (*P* for interaction = 0.020), and the results were stratified accordingly in Table 3, which shows that the association between higher screen time and higher total SDQ was particularly apparent in children with inadequate sleep duration.

**Table 3:** Stratified analysis of the association between screen time and strength and difficulties questionnaire scores by adequacy of sleep duration

Emotional and behavioral assessment	Screen time (h/d), *β* (95% CI)
Total SDQ score^a^	
Adequate sleep duration	0.25 (− 0.04, 0.54)
Inadequate sleep duration	0.65 (0.13, 1.18)*
Emotional symptoms scale^b^	
Adequate sleep duration	0.04 (− 0.06, 0.15)
Inadequate sleep duration	0.29 (0.10, 0.47)*
Conduct problems scale^b^	
Adequate sleep duration	0.05 (− 0.05, 0.15)
Inadequate sleep duration	0.23 (0.05, 0.42)*
Hyperactivity-inattention scale^b^	
Adequate sleep duration	0.09 (− 0.06, 0.24)
Inadequate sleep duration	0.03 (− 0.23, 0.28)
Peer problems scale^b^	
Adequate sleep duration	0.07 (− 0.02, 0.16)
Inadequate sleep duration	0.11 (− 0.06, 0.28)
Prosocial behaviors scale^c^	
Adequate sleep duration	− 0.07 (− 0.17, 0.04)
Inadequate sleep duration	− 0.02 (− 0.21, 0.18)

Linear regression models, with *β*-coefficients and 95% CI, were fitted to assess the associations between screen time and SDQ scores in the cohort stratified by adequacy of sleep duration (*P* for interaction = 0.020)

The model was fully adjusted for center size (according to the number of participants: < 200, 200–400, > 400), sex, age (in years), zBMI, physical activity (minutes/week), and exclusive breastfeeding during the first six months (no; yes), maternal and paternal BMI (kg/m^2^), educational level (primary or lower; secondary; academic/graduated), and socio-professional status (homemaker/student/retired/unemployed; employed)

Adequate sleep duration was defined as ranging between 10 and 14 hours/day for children under six years old, and between 9 and 12 hours/day for children over six years old (*n* = 1153). Sleeping less or more than these values was considered an inadequate sleep duration (*n* = 267)

^**a**^The total SDQ score considers all subscales with the exception of the prosocial scale, with higher scores indicating higher overall levels of emotional and behavioral difficulties. ^b^Higher scores indicate higher specific emotional and behavioral difficulties. ^c^Higher scores indicate higher prosocial capacities

* *P*-value < 0.05

*β (95% CI)* beta coefficients and 95% confidence intervals, *SDQ* strength and difficulties questionnaire, *BMI* body mass index, *zBMI* body mass index z-score

**Fig. 2:**
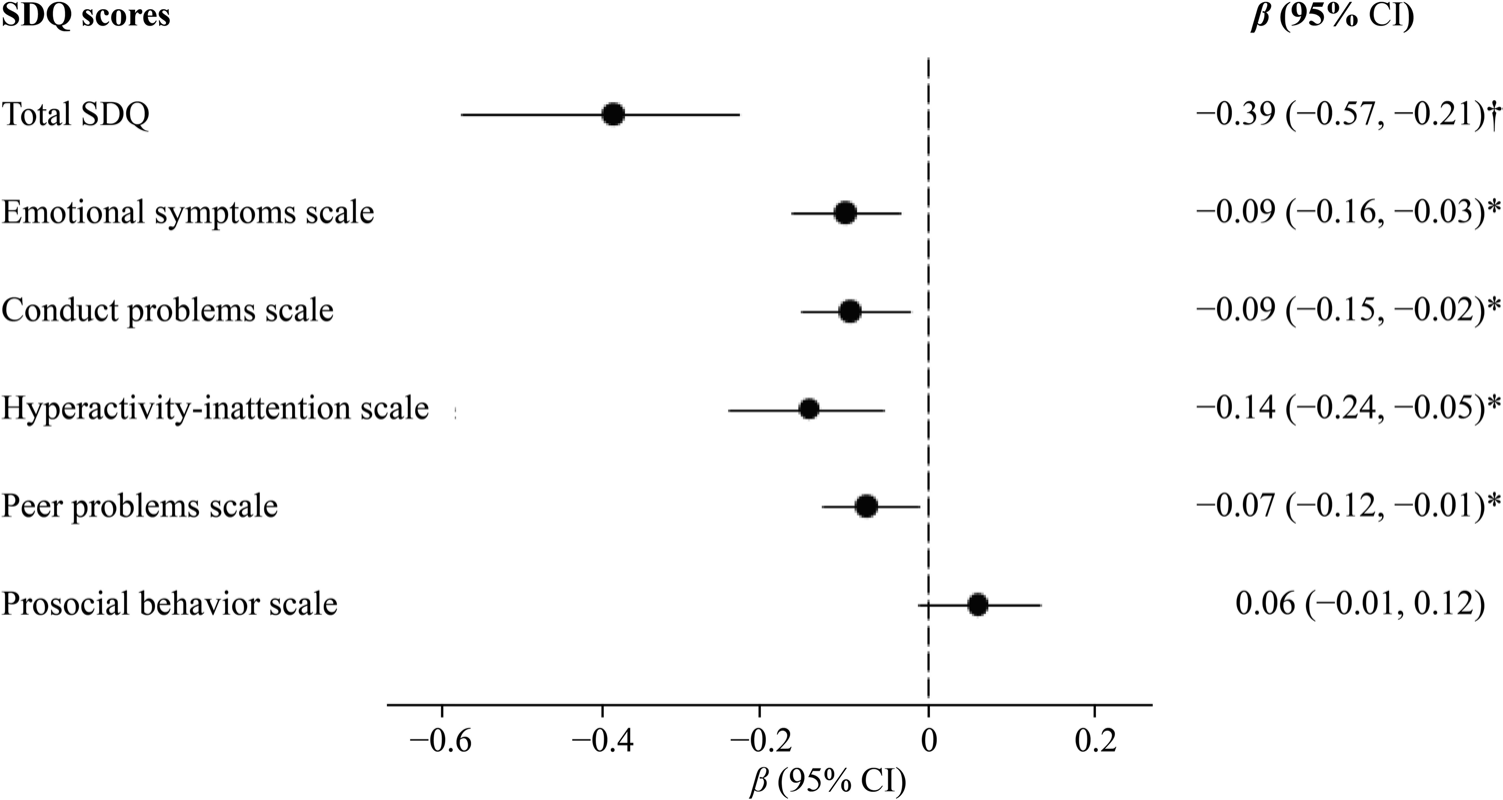
Isotemporal substitution of screen time (30 min/d) with sleep time on SDQ scores. Linear regression models were fitted to assess the associations of a simulation model that substitutes 30 min/d of screen time with an equivalent amount of sleep duration on all SDQ scores. Models adjusted for center size (according to the number of participants: < 250, 250–400, > 400), sex, age (in years), zBMI, physical activity (minutes/week), exclusive breastfeeding during the first six months (no; yes), and both maternal and paternal BMI (kg/m^2^), educational level (primary or lower; secondary; higher education), and socioprofessional status (homemaker/student/retired/unemployed; employed). * *P* value < 0.05, † *P* value < 0.001. *OR* odds ratio, *CI* confidence intervals, *SDQ* Strengths and Difficulties Questionnaire, *BMI* body mass index, *zBMI* body mass index z-score

Figure 2 shows that replacing 30 minutes from screen time to sleep was associated with lower scores on the total and subscales of the SDQ, except for the prosocial subscale.

As sensitivity analyses, Supplementary Fig. 2 shows the associations stratified by participants’ age, sex, weight status, adherence to the MedDiet, paternal education level and socioprofessional category, as well as by whether participants were assessed during the presence or absence of COVID-19 sanitation restrictions in Spain. In children with lower MedDiet adherence or whose fathers had higher education levels, positive associations were found between screen time and total SDQ. No additional significant interactions were observed. Supplementary Fig. 3 shows the Pearson correlation matrix between key study variables, including screen time, SDQ subscales, sleep duration, and age.

## Discussion

Our study revealed that greater screen time is associated with greater emotional and behavioral difficulties, whereas adherence to screen time recommendations is related to fewer psychosocial difficulties in children aged 3–6 years, particularly those with inadequate sleep duration. Additionally, our results suggest that substituting screen time with sleep duration may benefit children’s emotional and behavioral well-being.

However, recent meta-analyses examining the relationship between screen time and mental health have reported minimal effects or inconclusive results [11, 21]. These inconclusive findings likely reflect differences in study designs, sample sizes, age groups, cultural contexts, definitions of screen time (e.g., total time vs. type of use), measurement methods (self-reported vs. objective), and whether moderating factors such as sleep, diet, and parental involvement were considered. Additionally, the rapidly evolving nature of digital media, including the emergence of interactive platforms, educational apps, and diverse screen content, complicates generalization and comparisons across studies conducted in different time periods. Nevertheless, our findings align with those of several studies conducted in Europe, Asia, and North America that reported negative associations between screen time and children’s mental health [29–34].

Some mechanisms may explain the association between excessive use of screens and psychosocial issues. Physiologically, elevated screen time may disrupt neuroendocrine pathways (e.g., melatonin, cortisol, and insulin), leading to emotional dysregulation [35–37]. One hypothesis suggests that exposure to radiofrequency electromagnetic fields from wireless devices could impact the nervous system and contribute to emotional and behavioral problems [30, 35]. Lifestyle factors such as sedentary behaviors, sleep, diet, and parental supervision have also been suggested to contribute to the relationship between screen use and emotional and behavioral issues in childhood [9, 36, 38].

Sleep has received considerable attention in studies examining the associations between screen time and mental health [20, 21]. Our results showed that not only was longer sleep duration independently associated with fewer emotional and behavioral difficulties, which is consistent with prior literature [6, 18], but also a moderating effect of sleep duration was detected. Specifically, among children with inadequate sleep duration, greater screen time was linked to greater emotional and behavioral problems, suggesting a detrimental synergistic effect of greater screen time combined with reduced sleep on children’s psychosocial well-being. This finding aligns with previous research [18, 36, 39]; however, some studies have reported only small or negligible effects of sleep [21, 40], and others have emphasized the need for further research using longitudinal or repeated measures designs to better disentangle the complex relationship between screen time and sleep in children [21]. The results of our isotemporal substitution model further highlight the importance of sleep duration. This model assumes that replacing time from one activity to another can provide valuable insights into the behavioral trade-offs affecting health outcomes [41]. Replacing 30 min of daily screen time with sleep was associated with improved emotional and behavioral outcomes, highlighting the potential benefits of even small adjustments in daily routines to support psychosocial health. Physiological mechanisms may influence the relationships among screen time, sleep, and psychosocial health. Exposure to light-emitting screens—especially before bedtime—can delay sleep onset, reduce sleep quality, and suppress melatonin secretion [42–44]. Moreover, insufficient sleep duration has been associated with increased screen time in 17,7091 children [45], collectively suggesting a detrimental feedback loop between higher screen time and reduced sleep duration that disrupts circadian rhythms and may negatively impact mental health [46]. Nevertheless, this relationship has also been challenged in some studies involving children [40].

Our findings further indicate that greater screen time was particularly associated with greater emotional and behavioral issues in children with lower adherence to the Mediterranean diet. This suggests that inadequate adherence to nutrient-dense foods—rich in fruits, vegetables, whole grains, and healthy fats—may reduce the neuroprotective and anti-inflammatory benefits associated with better mental health [38, 47]. Interestingly, the association between screen time and total SDQ was stronger among children whose fathers had higher education levels, contrary to previous studies [38, 48]. This counterintuitive finding suggests that a higher parental educational level by itself may not protect against excessive screen exposure or its potential negative effects. This could reflect contextual challenges faced by parents, such as perceptions of educational screen benefits or difficulties in balancing work and caregiving [48]. Broader socioeconomic conditions, digital literacy, and the availability of time and resources to supervise or engage with children’s media use should also be considered relevant factors influencing these associations. In fact, co-using screens with caregivers and engaging with educational content can foster learning and positive interactions, whereas unsupervised use or exposure to inappropriate content is linked to poorer outcomes [49]. Furthermore, excessive parental screen time may contribute to reduced family engagement, parental stress, and family conflict, which in turn may impact children’s emotional and behavioral well-being [50].

Although the associations observed were statistically significant, the effect sizes reported in our study were small, indicating modest impacts at the individual level. However, in population-based research, even small effects can be meaningful, especially when exposures—such as screen time—are highly prevalent and accumulate over time. Nonetheless, we acknowledge the importance of avoiding overinterpretation of statistically significant yet potentially trivial effects [51].

This study has notable strengths, including a large sample size spanning seven Spanish cities, a standardized recruitment methodology, adjustments for multiple confounders, and several potential interactions. In addition, our results on psychosocial outcomes remained similar when the AAP or WHO compliance with screen time cutoffs was used, suggesting robustness.

However, several limitations should be considered. First, the cross-sectional design of this study does not allow the establishment of causal relationships. Second, the study was conducted in Spanish children, which could limit its generalizability. Third, several variables—including sleep duration, screen time, and behavioral outcomes—were based on parent reports, which are subject to recall and social desirability bias [52], potentially affecting the accuracy and validity of the reported data. For example, parents may overestimate sleep duration, as they may not accurately know when their child falls asleep, wakes up, or experiences night-time awakenings. Similarly, screen time may be underestimated due to concerns about excessive use. Fourth, although multiple confounders were considered, residual confounding cannot be ruled out. Fifth, while the present study focused on sleep duration, other relevant aspects of sleep—such as sleep timing and quality—were not evaluated because they were not assessed in the CORALS study. Sixth, when interpreting our findings, it is important to consider that although the WHO and AAP recommendations are widely used, they have also been criticized for relying partly on expert consensus rather than strong empirical evidence [11]. Seventh, although the COVID-19 pandemic did not have a moderating effect on our models, we cannot rule out its potential influence. Eighth, the study did not account for key contextual factors related to screen use, and the measure used was relatively coarse, as it did not capture the time-of-day children were exposed to screens, whether screens were used in bed, the types of screens used, or whether the activity was passive or interactive. Additionally, the content of screen exposure (e.g., educational vs. entertainment, violent vs. nonviolent) was not assessed, despite its potential relevance to mental health outcomes. Finally, the SDQ scores were unstandardized, and simple imputation methods were used for missing values on the covariates, which may have introduced minor bias.

In conclusion, the present study reinforces the growing body of evidence suggesting that greater screen time is associated with greater emotional and behavioral difficulties in children aged 3–6 years, particularly those with insufficient sleep duration. While our findings indicate that adherence to current screen time guidelines—less than two hours per day or under one hour for children under the age of five—may be beneficial for psychosocial health, the cross-sectional nature of the study limits causal interpretation. Additionally, replacing screen time with sleep may be associated with fewer emotional and behavioral difficulties. These findings support the importance of promoting balanced screen time and healthy sleep habits as part of broader public health initiatives to foster young children’s well-being in increasingly digital environments. Nevertheless, longitudinal and experimental studies are warranted to clarify causal pathways and better inform future evidence-based recommendations.

## Supplementary Information

Below is the link to the electronic supplementary material.

Supplementary file1 (DOCX 268 KB)

## Data Availability

The datasets generated and analyzed during the current study are not publicly available due to data regulations and for ethical reasons, considering that this information might compromise research participants’ consent because our participants gave their consent only for the use of their data by the original team of investigators. However, collaboration for data analyses can be requested by sending a letter to the CORALS Steering Committee (estudiocoral@corals.es). The request is then passed to all the members of the CORALS Steering Committee for deliberation.
