# Supplementary material for: Sleep duration moderates association between screen time and emotional and behavioural problems in young children
Source: World J Pediatr. 2025 Sep 23;21(11):1140–51. doi: 10.1007/s12519-025-00963-x (PMC12627151; doi:10.1007/s12519-025-00963-x)
Supplement: Supplementary file 1 — Supplementary file1 (DOCX 268 KB) [file 12519_2025_963_MOESM1_ESM.docx]

**Sleep duration moderates association between screen time and emotional and behavioural problems in young children**

**Supplementary Material**

[**Supplementary Methods** 2](#_Toc198549633)

[**The Strengths and Difficulties Questionnaire (SDQ)** 2](#_Toc198549634)

[**Covariates’ information** 2](#_Toc198549635)

[**Missing data and imputation** 2](#_Toc198549636)

[**Statistical analyses** 2](#_Toc198549637)

[*Isotemporal substitution analysis* 2](#_Toc198549638)

[**References** 3](#_Toc198549639)

[**Supplementary Figure 1.** Flowchart of CORALS participants analysed in the present study. 4](#_Toc198549640)

[**Supplementary** **Figure 2.** Associations between screen time and total SDQ score according to children’s age, sex, weight status, Mediterranean diet adherence, and parental educational level and socio-professional category. 5](#_Toc198549641)

[**Supplementary** **Figure 3.** Pearson correlation matrix between key study variables, including screen time, SDQ subscales, sleep duration, and age. 7](#_Toc198549642)

[**Supplementary Table 1.** Parental general characteristics according to the participant’s compliance with screen time recommendations. 8](#_Toc198549643)

# **Supplementary Methods**

## **The Strengths and Difficulties Questionnaire (SDQ)**

The SDQ contains 25 items grouped into five subscales (emotional symptoms, conduct problems, hyperactivity and inattention, peer problems, and prosocial behaviour) with three possible responses (0=“not true”, 1=“somewhat true”, and 2=“certainly true”). Each subscale is scored from 0 to 10 points. The total SDQ score (0-40 points) includes all subscales except the prosocial behaviour subscale. Higher scores on the total SDQ or on any of its four subscales are indicative of higher emotional and behavioural difficulties. Higher scores on the prosocial subscale are indicative of higher prosocial abilities.[1]

The clinical use of 16- and 17-points cut-offs was evaluated in a Spanish child and adolescent population comprising more than 6,000 participants, demonstrating their effectiveness in detecting psychiatric disorder diagnoses.[2]

## **Covariates’ information**

*Sociodemographic*: Sex (male; female) and age (in years) were obtained.

*Lifestyle behaviours*: Sleep duration (hours/day) was obtained and classified according to age (inadequate; could be adequate; adequate).[3] Physical activity (minutes/week) was obtained and categorized (healthy [≥120 minutes/week]; unhealthy [<120 minutes/week]).[4] Adherence to the Mediterranean diet (MedDiet) was obtained (range of 0-18 points).[5]

*Anthropometric:* Participants’ body weight (kg) and height (cm) were measured, and body mass index (BMI) was calculated. Based on the participants’ BMI (kg/m^2^), weight status was determined (underweight/normal weight [BMI<25]; overweight/obesity [BMI≥25].[6] Additionally, a BMI z-score (zBMI) was calculated using the standardized residual method, which controlled for sex and age.

*Early life factors*: Maternal weight gain during pregnancy (kg) was obtained. Birth weight (kg) was obtained and classified (low [<2.5kg]; normal [≥2.5 to <4kg]; high [≥4kg]).[7] Duration of breastfeeding (in months) was obtained and categorized (<2 years; ≥2 years). Exclusive breastfeeding was considered as exclusive breastfeeding during the first 6 months of life (yes; no).

*Parental factors*: Age (in years), self-reported BMI (kg/m^2^), weight status (underweight/normal weight; overweight/obesity), educational level (primary or lower; secondary; academic/graduated), and socio-professional status (homemaker/student/retired/unemployed; employed) were obtained.

## **Missing data and imputation**

All covariates had less than 2·5% missing data, except for paternal height which presented less than 9.8% missing data. Missing values were imputed by using the mean for numerical variables and the highest frequency for categorical variables.[8]

## **Statistical analyses**

### *Isotemporal substitution analysis*

In addition, a simulation model was fitted to substitute 30 minutes/day of screen time with an equivalent amount of sleep to assess its association with the total SDQ and its subscales. The theoretical isotemporal substitution effect was evaluated by simultaneously introducing both variables as continuous variables in the model. The β coefficients and 95% CIs for the substitution association were estimated from the differences in β coefficients, variances, and covariance.

## **References**

1. Goodman R. The strengths and difficulties questionnaire: A research note. J Child Psychol Psychiatry. 1997;38:581–6. doi: 10.1111/j.1469-7610.1997.tb01545.x

2. Español-Martín G, Pagerols M, Prat R, Rivas C, Sixto L, Valero S, et al. Strengths and Difficulties Questionnaire: Psychometric Properties and Normative Data for Spanish 5- to 17-Year-Olds. Assessment. 2020;28:1445–58. doi: 10.1177/1073191120918929

3. Hirshkowitz M, Whiton K, Albert SM, Alessi C, Bruni O, DonCarlos L, et al. National sleep foundation’s sleep time duration recommendations: Methodology and results summary. Sleep Health. 2015;1:40–3. doi: 10.1016/j.sleh.2014.12.010

4. WHO European Regional Obesity Report 2022. Copenhagen: WHO Regional Office for Europe; 2022. Licence: CC BY-NC-SA 3.0 IGO.

5. Garcidueñas-Fimbres TE, Paz-Graniel I, Gómez-Martínez C, Jurado-Castro JM, Leis R, Escribano J, et al. Associations Between Eating Speed, Diet Quality, Adiposity, and Cardiometabolic Risk Factors. J Pediatr. 2023;252:31-39.e1. doi: 10.1016/j.jpeds.2022.08.024

6. Cole TJ, Lobstein T. Extended international (IOTF) body mass index cut-offs for thinness, overweight and obesity. Pediatr Obes. 2012;7:284–94. doi: 10.1111/j.2047-6310.2012.00064.x

7. Yerushalmy J. The classification of newborn infants by birth weight and gestational age. J Pediatr. 1967;71:164–72. doi: 10.1016/s0022-3476(67)80067-2

8. Waal T de., Pannekoek Jeroen, Scholtus Sander. Handbook of data editing and imputation. John Wiley & Sons; 2011.

# **Supplementary Figure 1.** Flowchart of CORALS participants analysed in the present study.


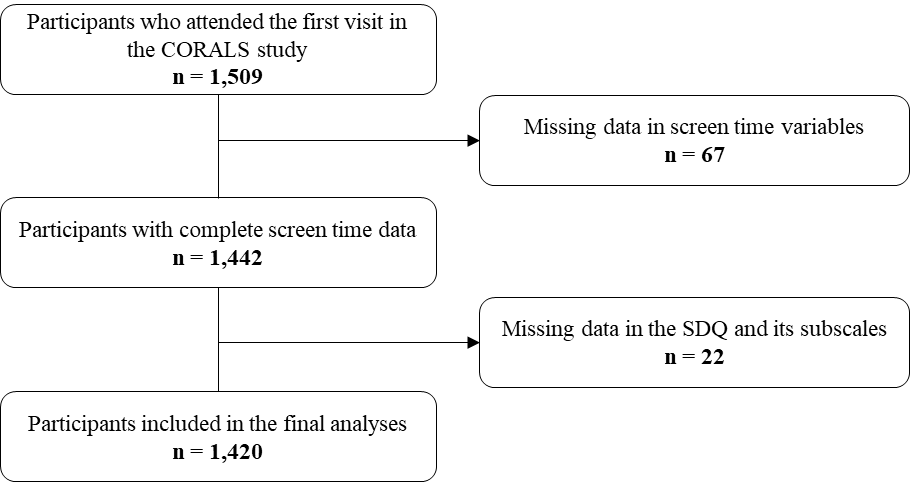


# **Supplementary** **Figure 2.** Associations between screen time and total SDQ score according to children’s age, sex, weight status, Mediterranean diet adherence, and parental educational level and socio-professional category.


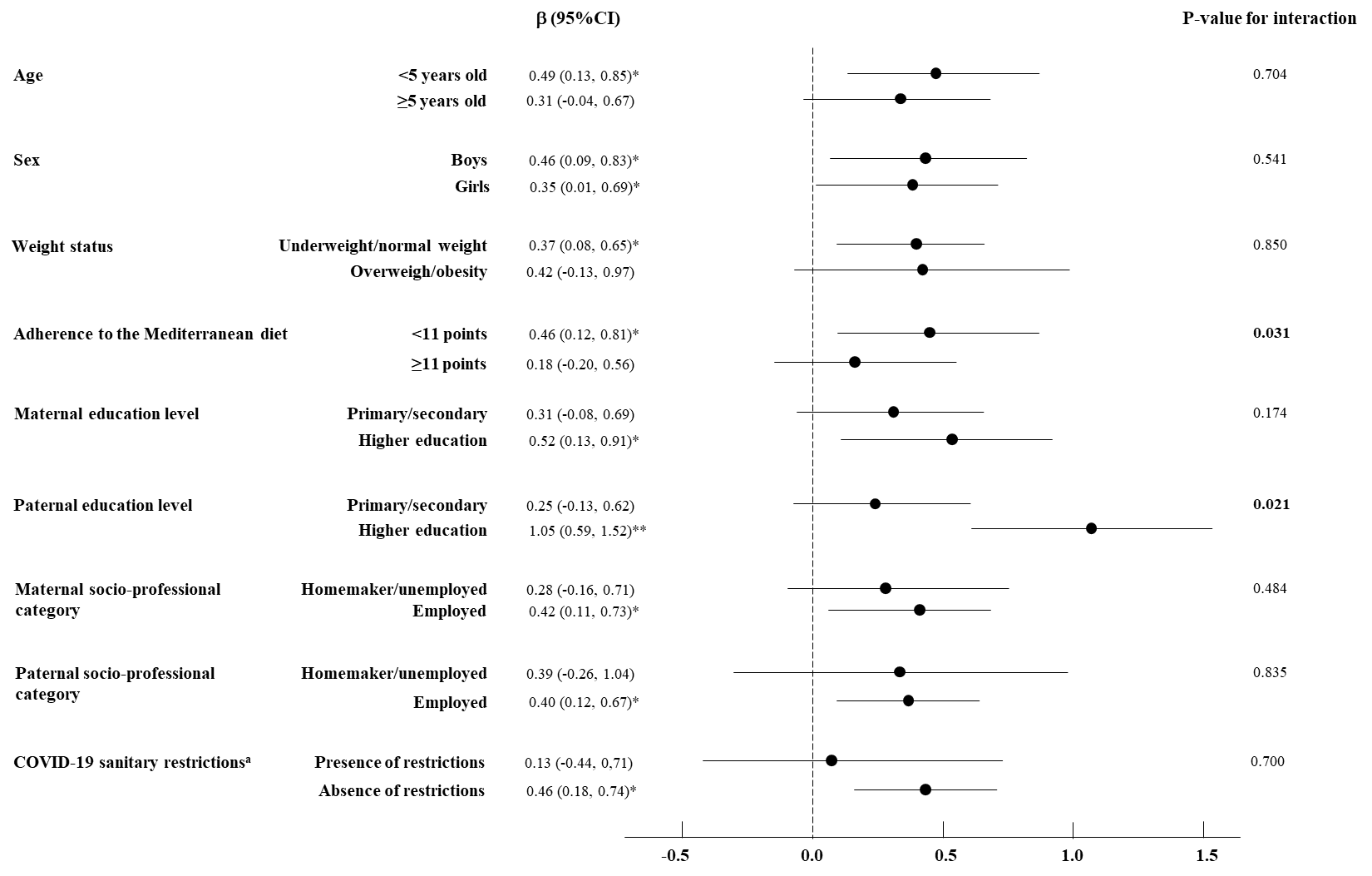


Linear regression models, with β-coefficients and 95%CI, were fitted to assess the associations between screen time and the total SDQ score according to potential interactions in the CORALS cohort. Potential interactions were assessed by using the likelihood ratio test.

Model adjusted for centre size (according to the number of participants: <200, 200-400, >400), sex, age (in years), zBMI, physical activity (minutes/week), exclusive breastfeeding during the first 6 months (no; yes), and both maternal and paternal BMI (kg/m2), educational level (primary or lower; secondary; academic/graduated), and socio-professional status (homemaker/student/retired/unemployed; employed). However, they were not adjusted for the variable of stratification in each case.

Higher scores in the total SDQ score indicate higher emotional and behavioural difficulties.

^a^ To assess the potential impact of the COVID-19 pandemic, participants were categorized according to their recruitment timing. Of the total sample, 1,122 children (79.01%) were recruited outside the period of major COVID-19 sanitary restrictions (March 14, 2020, to June 30, 2021), and 298 children (20.99%) were recruited during this period.

* *P*-value < 0.05 ** *P*-value < 0.001

# **Supplementary** **Figure 3.** Pearson correlation matrix between key study variables, including screen time, SDQ subscales, sleep duration, and age.


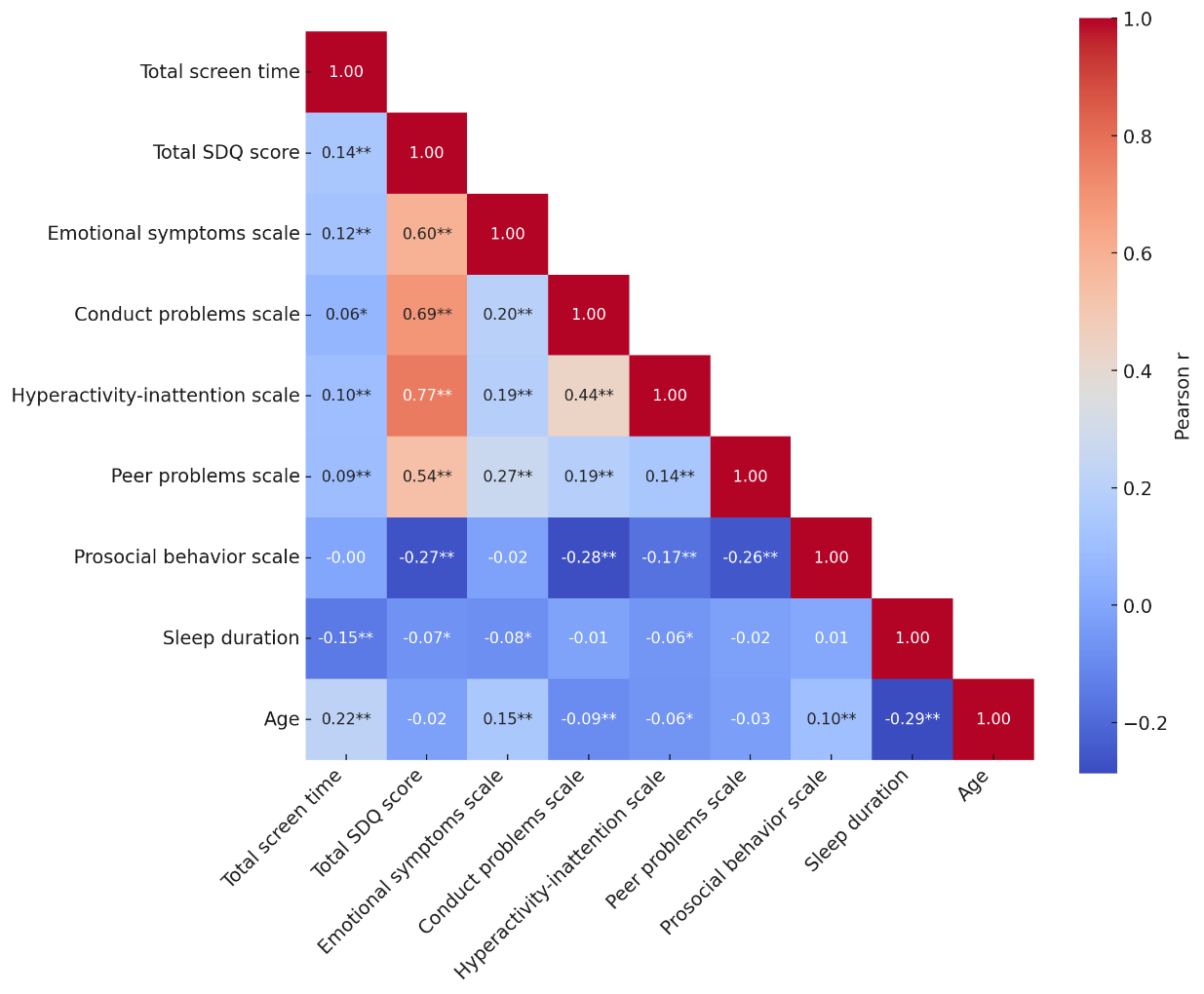


* *P*-value < 0.05 ** *P*-value < 0.001

| **Supplementary Table 1.** Parental general characteristics according to the participant’s compliance with screen time recommendations. | | | | |
| --- | --- | --- | --- | --- |
| **Variables** |  | **Categories of compliance with screen time recommendations (≤ 2 h/d)** | | |
|  | **All population**  *n* = 1420 | **Non-compliance**  *n* = 452  (>2 h/d) | **Compliance**  *n* = 968  (≤2 h/d) | *P*-value **^a^** |
| **Maternal factors** | | | | |
| Age (y) | 38.38 ± 5.00 | 38.30 ± 5.27 | 38.41 ± 4.82 | 0.706 |
| Mother weight gain during pregnancy, kg | 12.37 ± 5.28 | 12.18 ± 5.73 | 12.46 ± 5.06 | 0.351 |
| BMI, kg/m^2^ | 24.81 ± 4.76 | 25.70 ± 5.33 | 24.41 ± 4.42 | <0.001 |
| Weigh status |  |  |  | <0.001 |
| Underweight, normal weight, *n* (%) | 854 (60.14) | 236 (52.21) | 618 (63.84) |  |
| Overweight or obesity, *n* (%) | 566 (39.86) | 216 (47.79) | 350 (36.16) |  |
| Educational level |  |  |  | <0.001 |
| Primary or lower, *n* (%) | 132 (9.30) | 66 (14.60) | 66 (6.82) |  |
| Secondary, *n* (%) | 571 (40.21) | 209 (46.24) | 362 (37.40) |  |
| Academic or graduated, *n* (%) | 717 (50.49) | 177 (39.16) | 540 (55.79) |  |
| Socio-professional category, *n* (%) |  |  |  | 0.001 |
| Homemaker/student/retired/unemployed | 385 (27.11) | 148 (32.74) | 237 (24.48) |  |
| Employed | 1,035 (72.89) | 304 (67.26) | 731 (75.52) |  |
| Paternal factors | | | | |
| Age (y) | 40.63 ± 5.19 | 41.03 ± 5.24 | 40.44 ± 4.93 | 0.041 |
| BMI, kg/m^2^ | 26.89 ± 4.46 | 27.45 ± 4.41 | 26.63 ± 4.47 | 0.001 |
| Weigh status |  |  |  | <0.001 |
| Underweight or normal weight, *n* (%) | 465 (32.75) | 118 (26.11) | 347 (35.85) |  |
| Overweight or obesity, *n* (%) | 955 (67.25) | 334 (73.89) | 621 (64.15) |  |
| Educational level |  |  |  | <0.001 |
| Primary or lower, *n* (%) | 241 (16.97) | 119 (26.33) | 122 (12.60) |  |
| Secondary, *n* (%) | 698 (49.15) | 229 (50.66) | 469 (48.45) |  |
| Academic or graduated, *n* (%) | 481 (33.87) | 104 (23.01) | 377 (38.95) |  |
| Socio-professional category, *n* (%) |  |  |  | 0.044 |
| Homemaker/student/retired/unemployed | 174 (12.25) | 67 (14.82) | 107 (11.05) |  |
| Employed | 1246 (87.75) | 385 (85.18) | 861 (88.95) |  |
| *BMI* body mass index.  Data are expressed as mean ± SD for continuous variables or number (percentage) for categorical variables.  **^a^** *P*-values were calculated by t-test or chi squared tests. | | | | |
